# Enterococcus faecalis Bacteremia Following Transurethral Resection of the Prostate (TURP): Urinary Sepsis or Infective Endocarditis?

**DOI:** 10.7759/cureus.105053

**Published:** 2026-03-11

**Authors:** Carlos Robles, Jose Luis Donato

**Affiliations:** 1 General Medicine, Hospital Regional de Azuero Anita Moreno, Los Santos, PAN; 2 Cardiology, Hospital Regional de Azuero Anita Moreno, Los Santos, PAN

**Keywords:** bacteremia, enterococcus faecalis, infective endocarditis, sepsis, turp

## Abstract

*Enterococcus faecalis* is a gram-positive, facultative anaerobic bacterium that commonly colonizes the gastrointestinal and genitourinary tracts. Although frequently implicated in urinary tract infections and bacteremia, it is also a well-recognized cause of subacute infective endocarditis. We present a case of subacute infective endocarditis caused by *Enterococcus faecalis*, initially masked by presumed urinary sepsis in a patient who had undergone transurethral resection of the prostate (TURP) less than two months prior. The patient presented with four days of fever and generalized weakness, accompanied by clinical features of sepsis and urinalysis findings suggestive of urinary tract infection, indicating a possible urinary source. Empiric broad-spectrum antibiotic therapy was initiated for presumed urinary sepsis. However, persistent symptoms despite three days of treatment prompted further evaluation. Blood cultures grew *Enterococcus faecalis*, while urine cultures remained negative, raising suspicion for an alternative source of infection. After excluding intra-abdominal pathology through contrast-enhanced abdominopelvic computed tomography (CT), transthoracic echocardiography revealed mitral valve vegetation consistent with infective endocarditis. Targeted antimicrobial therapy with vancomycin for 14 days and ampicillin-sulbactam for 30 days resulted in complete clinical recovery and resolution of valvular vegetation without the need for surgical intervention.

This case highlights the importance of maintaining a high index of suspicion for infective endocarditis in patients with *Enterococcus faecalis* bacteremia, particularly when no clear infectious focus is identified. Additionally, it underscores the potential association between TURP-related bacteremia and subsequent endocarditis. These findings emphasize the need to reassess and optimize perioperative prophylactic strategies in patients undergoing urological procedures, taking into account regional antibiotic resistance, in order to reduce the risk of severe systemic complications such as infective endocarditis

## Introduction

*Enterococcus faecalis* is a gram-positive, facultative anaerobic coccus primarily found in the gastrointestinal tract [1]. This organism has intrinsic resistance to cephalosporins and benzylpenicillin (penicillin G), and some strains are even resistant to broad‑spectrum agents such as vancomycin or aminoglycosides [2]. Infections with this organism are associated with procedures involving the gastrointestinal and genitourinary tracts. Characteristic infections caused by *E. faecalis* include cholecystitis, intra‑abdominal collections, urinary tract infections, and endocarditis. Regarding the latter, *Enterococcus faecalis* typically causes subacute endocarditis, characterized by a delay of weeks to months from inoculation to the onset of initial symptoms [3,4]. Consequently, its clinical presentation is more insidious and prolonged compared with more common organisms such as Staphylococcus and Streptococcus. *E. faecalis* accounts for approximately 10% of all infective endocarditis cases [5]. According to the 2023 European Society of Cardiology (ESC) endocarditis guideline and the modified Duke criteria, Enterococci are considered typical organisms capable of causing this disease [6]. 

Because most patients with infective endocarditis present with nonspecific symptoms such as fever, malaise, and myalgias, and embolic and immunologic findings are present in only 5-10% of cases [2,6], diagnosis can be challenging. This difficulty can be greater when a patient has *E. faecalis* bacteremia following recent genitourinary surgery and has laboratory findings suggestive of a urinary tract infection. 

Transient bacteremia after TURP is a well‑described phenomenon that is clinically insignificant in most cases. The relationship between this transient bacteremia and the risk of endocarditis was examined by Stahl et al. (increased risk of infective endocarditis following transurethral resection of the prostate) [7], which found that the absolute risk was low. Among 25,781 post‑TURP patients, 901 developed bacteremia, and 44 of those patients developed infective endocarditis. In these 44 patients, 72.7% had endocarditis due to Enterococcus faecalis. Although the absolute risk was low, the study found that compared with age‑matched controls, post‑TURP patients had an eightfold increased risk of infective endocarditis within the first six postoperative months.

In this clinical case, we present a case report of a patient admitted with a working diagnosis of sepsis of urinary origin following a genitourinary procedure (TURP), who, in fact, had an underlying subacute *Enterococcus faecalis* endocarditis. Because of nonspecific symptoms and laboratory findings suggestive of a urinary infectious etiology, the initial diagnostic approach did not identify the patient’s infective endocarditis. We therefore aim to emphasize the importance of considering infective endocarditis in patients with *E. faecalis* bacteremia, even when a primary urinary source is apparent, taking recent TURP as a relevant risk factor for this disease.

## Case presentation

An 81-year-old male with a past medical history of hypertension, congestive heart failure, stage 3A chronic kidney disease, hypothyroidism, and benign prostatic hyperplasia status post transurethral resection of the prostate (TURP) one month prior (without immediate complications) was transferred from a local hospital to Hospital Regional Anita Moreno after a two-day hospitalization due to a four-day history of unquantified fever and generalized weakness. On physical examination, the patient was found disoriented with a Glasgow Coma Scale score of 14/15 (E4, M6, V4). Vital signs were as follows: blood pressure 154/85 mmHg, heart rate 69 bpm, respiratory rate 18 breaths per minute, temperature 37.1°C, and oxygen saturation 98% on room air. Cardiopulmonary examination revealed no significant abnormalities. The abdomen was soft, non-tender, and non-distended, with normal bowel sounds. A reducible right inguinal hernia was noted. The extremities were symmetrical, with no pathological findings, and the skin and soft tissues showed no signs suggestive of infection.

Initial laboratory studies demonstrated leukocytosis of 14,610/µL with neutrophilia (80.4%), hemoglobin 11.1 g/dL, hematocrit 34%, platelet count 249,000/µL, creatinine 1.50 mg/dL, N-terminal pro-brain natriuretic peptide 8224 pg/mL (NT-proBNP) and procalcitonin 1.55 ng/mL. Urinalysis revealed blood (++), 57.30 red blood cells per high-power field (HPF), 506.50 white blood cells per HPF, and yeast (2.10 per HPF). Blood and urine cultures were pending at admission.

The patient was admitted to the urology service with a diagnosis of complicated urinary tract infection (UTI) and was started on empiric intravenous antibiotic therapy with meropenem and vancomycin. While awaiting culture results, the patient showed no clinical improvement and developed chills, tremors, tachycardia, and tachypnea despite ongoing antibiotic therapy. Clinical findings were consistent with sepsis. Admission blood cultures revealed the presence of *Enterococcus faecalis*, resistant to fluoroquinolones and aminoglycosides but sensitive to ampicillin and vancomycin. Based on these results, antibiotic therapy was optimized to ampicillin-sulbactam and vancomycin, and meropenem was discontinued. Unexpectedly, the admission urine culture showed no growth at 48 hours, raising doubts about a urinary source of infection. A contrast-enhanced abdominopelvic CT scan was obtained to evaluate a possible intra-abdominal infectious source, guided by positive blood cultures for *E. faecalis*, to rule out intra-abdominal collections or other potential diagnoses that could explain the clinical presentation.

**Figure 1:**
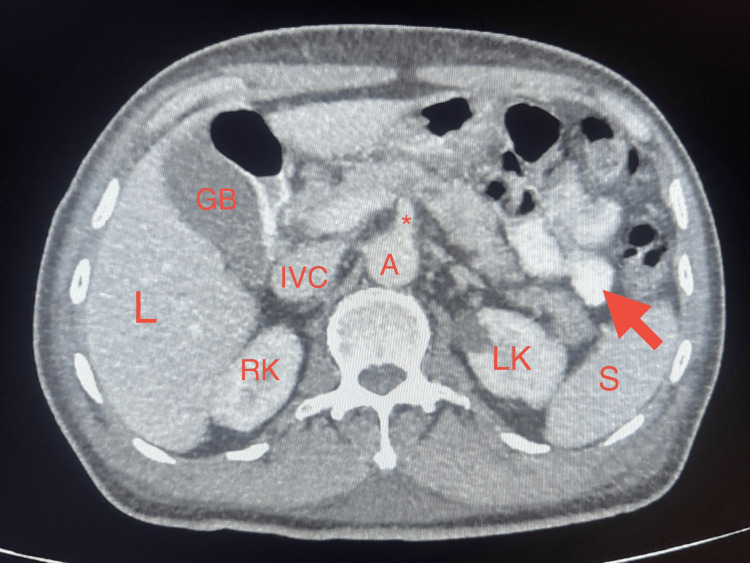
Contrast-enhanced abdominopelvic CT scan (axial abdominal view) Contrast-enhanced abdominopelvic CT scan (portal venous phase), axial view at the level of the superior mesenteric artery root, demonstrating normal distribution of vessels without evidence of intra-abdominal collection, abscess, or free fluid. The red arrow shows loops in the small intestine with contrast. GB=Gallblader, L=liver, RK: Right kidney, LK: Left kidney, IVC: Inferior vena cava, A: Abdominal aorta, *=Superior mesenteric artery, S: Spleen. Informed consent was given for the publication of the study.

**Figure 2:**
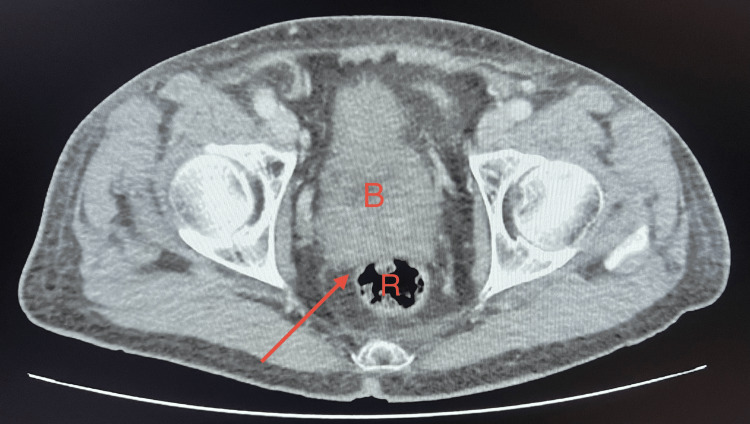
Contrast-enhanced abdominopelvic CT scan (axial pelvic view) Contrast-enhanced abdominopelvic CT scan (portal venous phase), axial pelvic view at the level of the rectovesical pouch, showing no pelvic collection, perirectal abscess, or dependent free fluid. The arrow shows the rectovesical space. B=Bladder, R: Rectum. Informed consent was given for the publication of the study.

**Figure 3:**
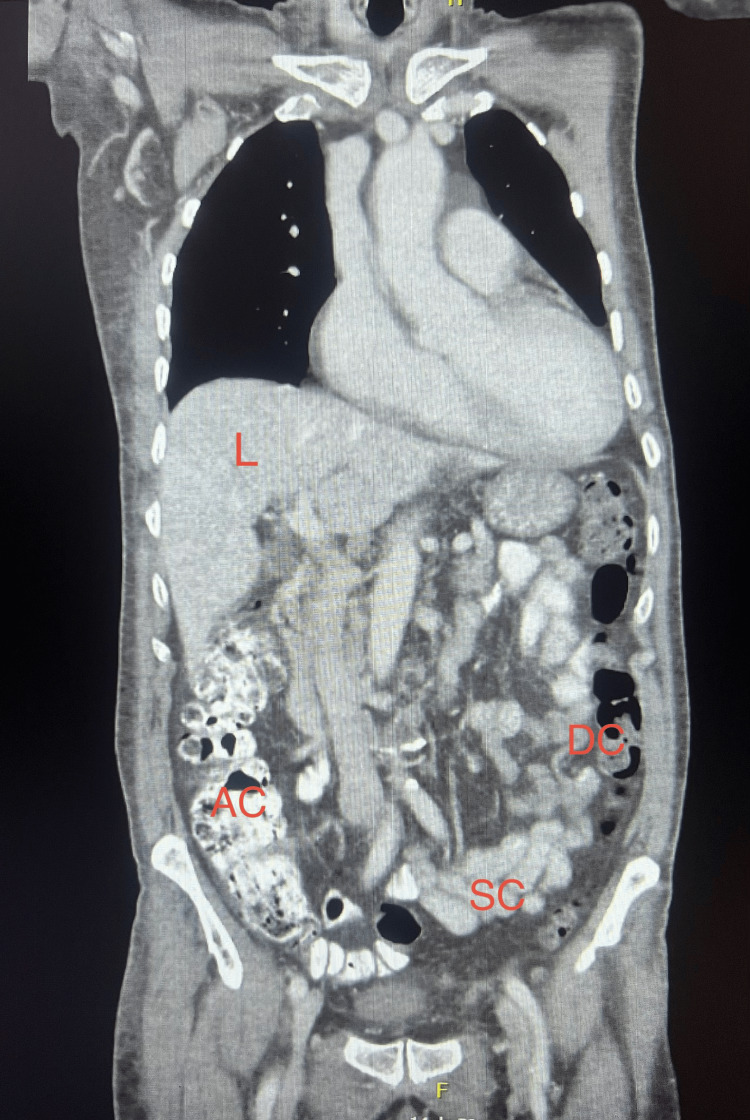
Coronal multiplanar reconstruction of contrast-enhanced abdominopelvic CT (coronal view) Coronal multiplanar reconstruction of contrast-enhanced abdominopelvic CT (portal venous phase), demonstrating absence of intra-abdominal or pelvic fluid collections or abscess formation across the coronal view. L=Liver, AC=Ascending colon, DC=Descending colon, SC=Sigmoid colon. Informed consent was given for the publication of the study.

**Figure 4:**
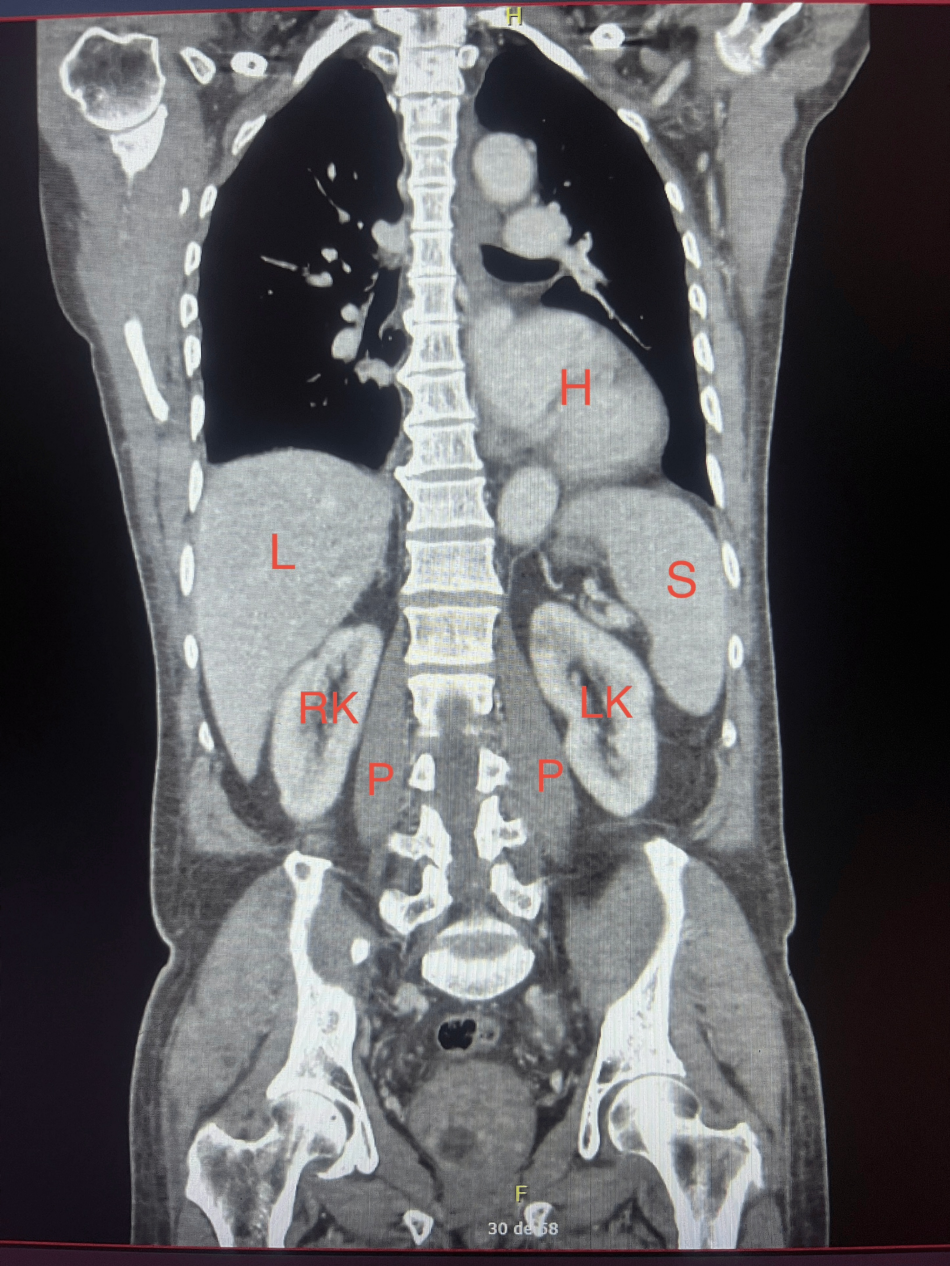
Coronal multiplanar reconstruction of contrast-enhanced abdominopelvic CT (retroperitoneal view) Coronal multiplanar reconstruction of contrast-enhanced abdominopelvic CT (portal venous phase), demonstrating absence of intra-abdominal or pelvic fluid collections or abscess formation on the retroperitoneum. H=Heart, =Liver, S=Spleen, RK=Right kidney, LK=Left kidney, P=Psoas muscle. Informed consent was given for the publication of the study.

Contrast-enhanced abdominopelvic CT revealed no gastrointestinal pathological findings that could explain the patient’s septic state (Figures 1-4). The previously known right inguinal hernia was confirmed. At the pelvic level, an enlarged prostate measuring 6.3 × 5.6 × 5.0 cm, with an estimated weight of 97 grams, was observed.

**Figure 5:**
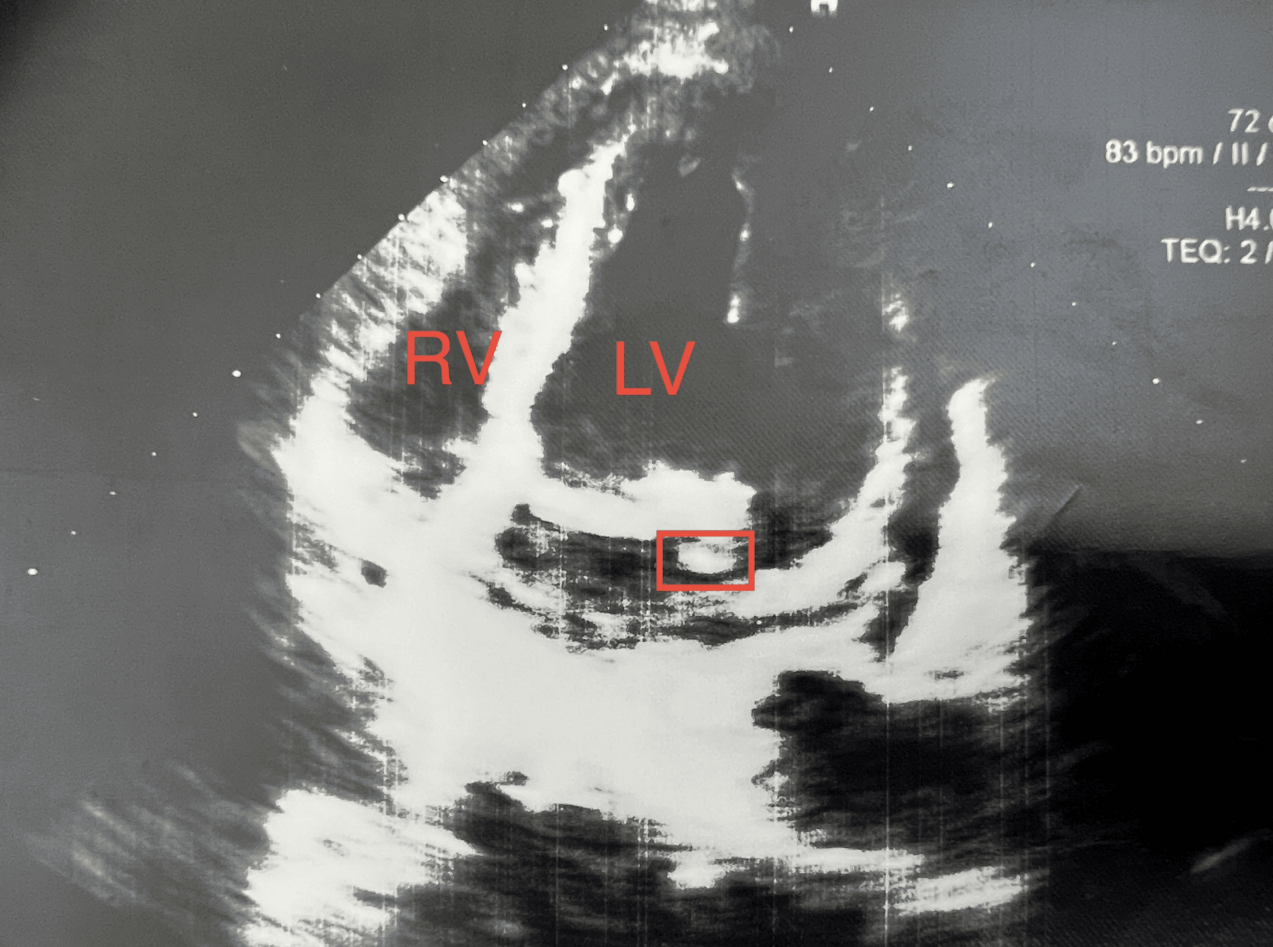
Diagnostic ecocardiography The ecocardiography performed on the patient shows a 6 × 3 mm vegetation (highlighted by the red square) was visualized on the posterolateral leaflet of the mitral valve. LV=Left ventricle, RV=Right ventricle. Informed consent was given for the publication of the study.

Incidental findings at the lower thoracic level included cardiomegaly predominantly due to left ventricular enlargement, a small pericardial effusion, bilateral pleural effusions, and passive atelectasis at the lung bases. In the absence of an identifiable gastrointestinal infectious source, a literature review was conducted, revealing an association between *E. faecalis* bacteremia and infective endocarditis, as well as a reported relationship between TURP procedures and subsequent infective endocarditis. A transthoracic echocardiography was positive for infective endocarditis (Figure 5). At the level of the mitral valve, moderate regurgitation was observed. The anteromedial leaflet tip appeared thickened, and a 6 × 3 mm mobile vegetation was identified on the posterolateral leaflet.

At this stage, the patient fulfilled two major Duke criteria: (1) echocardiographic findings characteristic of infective endocarditis and (2) two sets of positive blood cultures consistent with infective endocarditis (Table 1). Additionally, one minor criterion was present: fever greater than 38°C. Therefore, a definitive diagnosis of infective endocarditis was established according to the modified Duke criteria. Following confirmation of the diagnosis, an infectious disease consultation was requested for further management and follow-up. Serial laboratory monitoring every 72 hours was initiated, including complete blood count (CBC), renal function panel, electrolytes, and procalcitonin levels.

**Table 1:** 2023 modified Duke criteria for infective endocarditis The patient fulfilled two major criteria and one minor criterion, thereby establishing the diagnosis of infective endocarditis. Adapted from Delgado et al. [6].

Major criteria
Blood culture positive for IE
Typical microorganisms consistent with IE from two separate blood cultures: *Viridans streptococci*, *Streptococcus bovis*, HACEK group, *Staphylococcus aureus*; or community-acquired enterococci in the absence of a primary focus, or microorganisms consistent with IE from persistently positive blood cultures defined as follows: at least 2 positive cultures of blood samples drawn >12 h apart or all 3 or a majority of ≥4 separate cultures of blood (with first and last sample drawn at least 1 h apart)
Single positive blood culture for *Coxiella burnetii* or anti–phase 1 IgG antibody titer ≥1:800
Evidence of endocardial involvement
Echocardiogram positive for IE (TEE recommended for patients with prosthetic valves, rated at least possible IE by clinical criteria, or complicated IE [paravalvular abscess]; TTE as first test in other patients) defined as follows: oscillating intracardiac mass on valve or supporting structures, in the path of regurgitant jets, or on implanted material in the absence of an alternative anatomic explanation; abscess; or new partial dehiscence of prosthetic valve or new valvular regurgitation (worsening or changing or pre-existing murmur not sufficient)
Minor criteria
Predisposition, predisposing heart condition, or IDU
Fever, temperature >38°C
Vascular phenomena, major arterial emboli, septic pulmonary infarcts, mycotic aneurysm, intracranial hemorrhage, conjunctival hemorrhages, and Janeway lesions
Immunological phenomena: glomerulonephritis, Osler nodes, Roth spots, and rheumatoid factor
Microbiological evidence: positive blood culture, but does not meet a major criterion as noted above (excludes single positive cultures for coagulase-negative staphylococci and organisms that do not cause endocarditis) or serological evidence of active infection with an organism consistent with IE.

In the subsequent days, the patient demonstrated progressive clinical improvement, with resolution of fever and no further clinical signs of sepsis, eventually becoming asymptomatic. He was evaluated by the infectious disease specialist, who recommended continuing ampicillin/sulbactam for a total of four weeks and discontinuing vancomycin on day 14. Follow-up echocardiographic studies showed complete resolution of the vegetation, with no evidence of residual valvular inflammation, no mitral regurgitation, and a left ventricular ejection fraction (LVEF) of 50%.

Serial laboratory studies demonstrated progressive improvement, including normalization of inflammatory markers and NT-proBNP levels (Table 2). The last laboratory results prior to discharge showed: leukocytes 4.95 ×10³/µL, neutrophils 64.7%, lymphocytes 23.8%, hemoglobin 11.0 g/dL, hematocrit 33.9%, and platelets 165,000/µL. After completing one month of antibiotic therapy with ampicillin, the patient remained afebrile and asymptomatic. This confirmed a favorable response to antimicrobial therapy, with full clinical resolution and no need for surgical intervention. The patient was subsequently discharged.

**Table 2:** Comparative laboratory results from admission to discharge

Laboratory Parameter	Admission Value	Discharge Value	Units	Reference Range
Leukocytes	14,610	4.95	×10³/µL	4.0–10.0 ×10³/µL
Neutrophils	80.4	64.7	%	40–75%
Hemoglobin	11.1	11.0	g/dL	13–17 g/dL
Hematocrit	34	33.9	%	40–52%
Platelets	249,000	165,000	/µL	150,000–400,000/µL
Procalcitonin	1.55	<0.05	ng/mL	<0.05 ng/mL
NT-proBNP	8224	792	pg/mL	<1800 pg/mL (<75 years)

## Discussion

Due to the patient’s nonspecific symptoms, recent urological surgical history, and urinalysis findings suggestive of infection, a urinary tract infection was initially considered the most likely diagnosis of the clinical presentation. One of the main challenges in this case was the patient’s advanced age. In older adults, lower urinary tract infections often do not present with classic urinary symptoms, but rather with nonspecific manifestations such as disorientation, delirium, or anorexia [8]. Therefore, the clinical and laboratory findings at admission were initially consistent with a urinary tract infection. However, culture results raised suspicion for an alternative diagnosis: positive blood cultures for *Enterococcus faecalis* in the setting of a negative urine culture. After a contrast-enhanced abdominopelvic CT scan failed to identify any intra-abdominal pathology that could explain the septic presentation, attention shifted toward a cardiac etiology. This suspicion was confirmed by echocardiography, which established the diagnosis of infective endocarditis. The patient was successfully treated with antibiotic therapy alone, consisting of ampicillin/sulbactam for one month and vancomycin for 14 days.

In the study by Andersen et al. [9], the prevalence of infective endocarditis in patients with *E. faecalis* bacteremia was evaluated. The methodology consisted of performing echocardiography in all patients with positive blood cultures for gram-positive cocci within the past six months. In the case of *E. faecalis*, the study showed that 33% of the patients with *Enterococcus faecalis* bacteremia developed infective endocarditis [9]. Therefore, *E. faecalis* bacteremia should serve as a clinical red flag for possible infective endocarditis, particularly in patients without a clearly identifiable infectious focus. This is especially important given that infective endocarditis is associated with high morbidity and mortality, with mortality rates reaching up to 30% within the first 30 days after symptom onset [10].

In the post-TURP setting, the association between procedure-related bacteremia and the subsequent development of infective endocarditis has been described [7]. Therefore, clinicians should maintain a high index of suspicion for infective endocarditis in cases of *E. faecalis* bacteremia following TURP. Furthermore, this association highlights the importance of optimizing prophylactic strategies in patients undergoing such procedures, as TURP may represent a potential risk factor for infective endocarditis.

Regarding perioperative prophylaxis during the prior TURP, the patient received intravenous ciprofloxacin, which is included among recommended prophylactic regimens for urological procedures. Although ciprofloxacin is not the first-line agent for *Enterococcus faecalis*, it has activity against this organism [11]. In this case, blood cultures grew *E. faecalis* resistant to quinolones and aminoglycosides. Fluoroquinolone resistance is a growing concern in Panama. This resistance pattern may have contributed to inadequate perioperative coverage, potentially facilitating bacteremia and subsequent endocarditis. While specific resistance data for *E. faecalis* are limited, studies evaluating other gastrointestinal pathogens, such as *Escherichia coli*, have reported resistance rates that could reach up to 76% [12].

Finally, it is worth noting that the 2023 modified Duke criteria include *Enterococcus faecalis* among the typical microorganisms associated with infective endocarditis [6]. However, it must be emphasized that even though the Duke criteria were developed as a diagnostic tool, strict reliance on these criteria may result in missed diagnoses of infective endocarditis. In this case, the patient did not present with a new murmur, vascular or immunologic phenomena, or known valvular disease predisposing to endocarditis. Clinical judgment remains the cornerstone of diagnosis.

## Conclusions

This case highlights the importance of maintaining a high index of suspicion for infective endocarditis in patients with *Enterococcus faecalis* bacteremia, particularly when the clinical presentation is nonspecific. Misattribution to an alternative infectious focus may delay diagnosis and appropriate treatment, thereby increasing the risk of complications. Additionally, this case underscores the relevance of considering TURP as a potential risk factor for infective endocarditis and reinforces the need to strengthen diagnostic vigilance and prophylactic strategies, taking into account regional antibiotic resistance, in this patient population.
